# Nanometer Resolution Structure‐Emission Correlation of Individual Quantum Emitters via Enhanced Cathodoluminescence in Twisted Hexagonal Boron Nitride

**DOI:** 10.1002/adma.202501611

**Published:** 2025-07-24

**Authors:** Hanyu Hou, Muchuan Hua, Venkata Surya Chaitanya Kolluru, Wei‐Ying Chen, Kaijun Yin, Pinak Tripathi, Maria K.Y. Chan, Benjamin T. Diroll, Thomas E. Gage, Jian‐Min Zuo, Jianguo Wen

**Affiliations:** ^1^ Center for Nanoscale Materials Argonne National Laboratory 9700 S. Cass Avenue Lemont IL 60439 USA; ^2^ Department of Materials Science and Engineering Materials Research Laboratory University of Illinois Urbana Champaign 1304 W. Green St. MC 246 Urbana IL 61801 USA; ^3^ Nuclear Science and Engineering Argonne National Laboratory 9700 S. Cass Avenue Lemont IL 60439 USA

**Keywords:** 2D material, atomic structure, cathodoluminescence, hexagonal boron nitride, quantum emitter, quantum information science, single photon emitter, twisted interface

## Abstract

Understanding the atomic structure of quantum emitters, often originating from point defects or impuritie, is essential for designing and optimizing materials for quantum technologies such as quantum computing, communication, and sensing. Despite the availability of atomic‐resolution scanning transmission electron microscopy and nanoscale cathodoluminescence microscopy, experimentally determining the atomic structure of individual emitters is challenging due to the conflicting needs for thick samples to generate strong cathodoluminescence signals and thin samples for structural analysis. To overcome this challenge, significantly enhanced cathodoluminescence at twisted interfaces is leveraged to achieve sub‐nanometer localization precision for the first time in mapping individual quantum emitters in carbon‐implanted hexagonal boron nitride. This unprecedent spatial sensitivity, together with correlative electron energy loss spectroscopy quantitative scanning transmission electron microscopy imaging, and first principles density functional theory calculations, enables the identification of the atomic structure of the 440 nm blue emitter in hexagonal boron nitride as a substituted vertical carbon dimer. Building on the atomic structure insights, nanoscale spatially precise creation of blue emitters is demonstrated by electron beam irradiation of carbon‐coated hexagonal boron nitride. This advancement in correlating atomic structures with optical properties lays the foundation for a deeper understanding and precise engineering of quantum emitters, significantly advancing the development of cutting‐edge quantum information technologies.

## Graphical abstract

Nanometer‐resolution mapping of individual quantum emitters in hexagonal boron nitride is achieved, allowing us to identify the 440 nm blue emitter as a substituted vertical carbon dimer. Leveraging this atomic‐level insight, the deterministic creation of isolated single 440 nm blue emitters is demonstrated through localized electron beam irradiation of carbon‐coated hexagonal boron nitride.

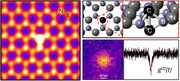

## 1 Introduction

A deep understanding of solid‐state quantum emitters (QEs), especially those based on atomic‐scale defects, is essential for advancing quantum information science, including applications in quantum computing, communication, and sensing.^[^ ^1^, ^2^ ^]^ Since the optical and electronic properties of QEs are governed by their atomic structures, correlating these atomic structures with optical properties is crucial. Such insights not only expand fundamental knowledge but also enable the intentional creation and manipulation of QEs with tailored properties. To date, analyses of atomic structures in quantum emitters have primarily relied on density functional theory (DFT) calculations, aligning computational predictions with experimental emission data.^[^ ^3^, ^4^ ^]^ However, validating these models and discovering new QEs requires experimental approaches to establish direct structure‐property relationships at the level of individual single‐photon sources. Achieving this goal depends on two key elements: the precise localization of individual active emitters and ensuring that the area is suitable for further atomic‐scale characterization.

Far‐field confocal photoluminescence (PL) is widely used to study QE optical properties but lacks the spatial resolution to pinpoint individual emitters at lattice sites.^[^ ^5^ ^]^ This limitation makes it challenging to precisely identify the corresponding defect structure responsible for observed single photon emission among many possible defect configurations. Cathodoluminescence (CL) offers a powerful alternate luminescence technique which uses electrons as an excitation source. By focusing the electron beam and thus localizing excited charge carriers, CL can overcome the optical diffraction limit of far field optical microscopy and provide luminescence information with a spatial resolution that is orders of magnitude better.^[^ ^6^, ^7^ ^]^ With recent advances in aberration‐corrected electron optics, scanning transmission electron microscopes (STEM) can now produce electron probes smaller than 0.1 nm, providing a highly accessible means of achieving atomic‐scale characterizations of structural, chemical, and electronic information. Therefore, integrating atomic‐resolution STEM with nanoscale CL microscopy (STEM‐CL) provides new exciting opportunities for studying the structure‐property of QEs,^[^ ^7^ ^]^ especially since the combination simultaneously enables the enhanced spatial resolution CL microscopy of active emitters and atomic resolution structural characterization.

Mapping active QEs in hexagonal boron nitride (hBN) with CL has so far been largely limited to relatively thick samples (>100 nm),^[^ ^8^ ^]^ as a large electron‐sample interaction volume has so far been necessary to produce sufficient excited electrons for CL emissions. Using STEM‐CL, Hayee et al. identified multiple active QEs in hBN nanoflakes with a spatial resolution of ≈50 nm.^[^ ^9^ ^]^ These pioneering STEM‐CL works have made significant contributions to advancing our understanding of the optical properties of quantum emitters at the nanoscale.^[^ ^7^, ^9^ ^]^ However, the sample thickness restricts further structural and chemical characterization of QEs using STEM. Atomically thin or few‐layered 2D materials, such as transition metal dichalcogenides (TMDs) and hBN, offer a simplified platform for exploring structure‐property relationships through STEM‐CL, provided that a sufficient CL signal can be reliably collected. By significantly increasing the probe size, sufficient STEM‐CL signals have been successfully obtained from atomically thin TMD samples. However, this approach sacrifices spatial resolution, limiting it to ≈100 nm.^[^ ^10^ ^]^ While the larger probe size enhances CL emission, the resulting resolution is inadequate for accurately localizing emitters.

Recently, a significant enhancement in CL at the twisted hBN interface was reported using scanning electron microscopy‐CL (SEM‐CL).^[^ ^11^ ^]^ By leveraging enhanced CL at a twisted interface, we significantly reduced the interaction volume, achieving high‐spatial‐resolution CL microscopy with a resolution of ≈10 nm. This capability allows for nanoscale CL mapping to localize individual QEs with sub‐nanometer localization precision. Using this approach, we successfully carried out correlative characterization of blue emitters with a zero phonon line (ZPL) of 440 nm^[^ ^12^, ^13^ ^]^ in thin twisted hBN samples. Through STEM chemical analysis and atomic resolution imaging, we determined the atomic structure of these blue emitters to be a split interstitial defect as vertically aligned carbon dimer (VACD), which is further confirmed with defect levels calculated by DFT. Based on this knowledge, we then developed a lithographic approach for the creation of blue emitters.

## 2 Results

### 2.1 STEM‐CL Enhancement at Twisted Interface in hBN

**Figure 1:**
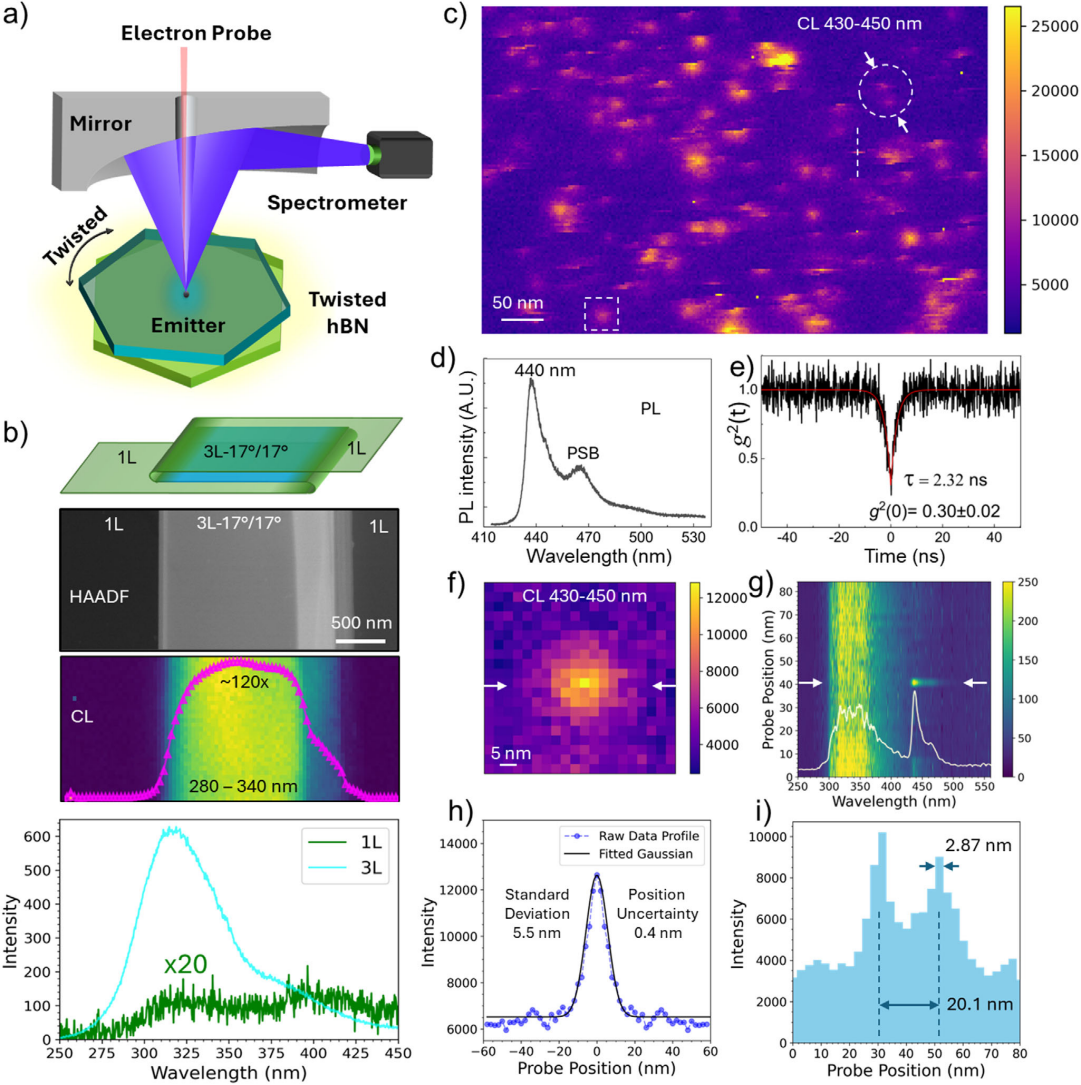
High spatial resolution CL mapping of QEs enabled by twisted interfaces. a) Schematic setup for simultaneous STEM imaging and CL microscopy. b) Schematic diagram (upper) of twisted hBN with a twisted angle of 17° in the folded area, HAADF image and CL intensity profile (middle), and CL spectra from 1L and 3L‐hBN (lower). The CL intensity profile shows ≈120 times CL enhancement in twisted 3L‐hBN. Note the intensity of the CL spectrum from 1L‐hBN (lower) is multiplied by 20 times. c) CL mapping (430–450 nm) of 440 nm blue emitters from a different carbon‐ion irradiated hBN. d) PL spectrum of a 440 nm blue emitter. e) g^(2)^(t) measurements showing the single‐photon emission of the 440 nm emitter, with a lifetime of 2.32 ns. f) Zoom‐in CL map of a single 440 nm blue emitter from the white square box in c). g) CL spectral line scan along the white line in c) showing another 440 nm emitter. The inset shows the CL spectrum of the blue emitter at the location indicated by the two arrows in panel g), corresponding to a single 2.8 nm pixel containing only one emitter. h) Gaussian fitting of another emitter (Figure S4, Supporting Information) showing a standard deviation of 5.5 nm with a position uncertainty of 0.4 nm. The intensity profile is cut along two arrows in Figure S4b (Supporting Information). i) The CL intensity profiles of the two blue emitters circled in panel c), taken along the two white arrows, are clearly distinguished.

**Figure** **1**a shows a schematic diagram of our STEM‐CL system, in which the TEM sample is decoupled from the parabolic mirror. The emitted photons are reflected by the parabolic mirror and collected by a spectrometer through an optical fiber. Figure 1b shows a high‐angle annular dark‐field (HAADF) image (top), corresponding STEM‐CL intensity map and CL intensity profile (middle), and CL spectra from 1L and 3L‐hBN (lower), revealing a significant enhancement of the CL signal from ultraviolet (UV) emitters. This enhancement is observed in the folded area of a freestanding exfoliated pristine hBN flake (Detailed sample information can be found in Table S1, Supporting Information). This UV emission features a major broad peak at 320 nm (3.9 eV) (lower part in Figure 1b), attributed to the intrinsic emitters with high density in the sample.^[^ ^14^, ^15^, ^16^, ^17^ ^]^ To investigate whether the broad peak convolved with other UV emission lines such as the 300 nm ZPL reported in previous literature, we performed cryogenic CL measurement at −186 °C, and the 300 nm feature in this sample is weak, but observable as shown in Figure S1 (Supporting Information).^[^ ^14^, ^15^, ^16^, ^17^ ^]^ Twisting angles in the double twisted interface are measured as high twisting angles of 17°/17° (Figure S2, Supporting Information). Compared to the UV emission from the triple‐layer, the CL signal from the single‐layer is nearly invisible as the average signal is approaching 0 (lower part of Figure 1b). EELS measurements (Figure S2, Supporting Information) indicate a thickness of 30 nm for the unfolded area and 90 nm for the twisted triple‐layer. Therefore, collecting sufficient CL signals from untwisted hBN at 30 nm or thinner is extremely challenging with our CL system. The CL signal from UV emission in the twisted triple‐layer (with two twisted interfaces) is over 120 times stronger than that from the single layer, as shown in Figure 1b. The observed CL enhancement varies from ×10 to ×120 depending on the twist angle, the quality of the interface contact, and the number of twisted interfaces. The gradual decrease in CL intensity which is observed at the right edge of the folded hBN is due to imperfect interfacial contact between the two hBN layers. This observation indicates that an interfacial amplified CL emission at twist hBN works for both SEM‐CL and STEM‐CL.^[^ ^11^ ^]^ One potential mechanism for the observed enhancement is the modification of the electronic band structure and density of states (DOS) in the moiré pattern.^[^ ^11^, ^18^, ^19^, ^20^ ^]^

### 2.2 Nanometer Resolution STEM‐CL Mapping of Individual QEs

By leveraging the enhanced CL signal, individual emitters can be detected using hyperspectral CL mapping with a sub‐nanometer probe, although the degree of CL enhancement may vary by emitter type and is not necessarily as high as the 120 × observed for UV emitters. Figure 1c presents a hyperspectral CL mapping from a twisted area in a different carbon‐ion irradiated hBN (sample details in Table S1, Supporting Information). The integrated CL spectrum (Figure S3a, Supporting Information) from the mapping area reveals blue emissions at 440 nm in addition to UV emissions (280–350 nm). The blue emitter with a ZPL at 440 nm was first observed by electron beam irradiation on hBN.^[^ ^12^, ^13^, ^21^, ^22^ ^]^ This emitter exhibits exceptional optical properties, including long survival time, high stability, and single‐photon emission.^[^ ^21^ ^]^ We also confirmed that the blue emitters in our carbon‐ion irradiated hBN samples exhibit single‐photon emission by measuring the second‐order photon correlation, g^(2)^(t). This is supported by the PL spectrum shown in Figure 1d and the g^(2)^(t) measurement displaying a clear dip at t = 0 in Figure 1e. In Figure 1c, numerous isolated round bright spots (such as the one shown in Figure 1f) corresponding to individual blue emitters are visible, along with several brighter spots of irregular shapes, representing clusters of blue emitters. Figure 1g further highlights the 440 nm blue emission through a CL spectral line scan along the white dash line in Figure 1c, with the inset CL spectrum showing the corresponding 440 nm emission peak. Unlike the relatively sparse distribution of blue emitters, UV emitters are more densely and uniformly distributed, as shown in the overlapping map (Figure S3d, Supporting Information). Their significantly higher density exceeds the resolution limit of current CL mapping, making it difficult to resolve individual UV emitters.

Figure 1f shows a single 440 nm emitter (from the white box in Figure 1c), with the brightest central emission within a single pixel (2.8 nm), enabling precise determination of the emitter's position within a 2.8 nm diameter based on the scanning steps. Alternatively, super‐resolution microscopy can be employed to localize the emitter by fitting the point spread function (PSF) to its center of mass. For another mapping with a scanning step size of 2 nm (Figure S4, Supporting Information), the fitted Gaussian distribution closely matches the emission profile, yielding a standard deviation (σ) of 5.5 nm and position uncertainty smaller than 0.43 nm (Figure 1h). Thus, the calculated spatial resolution is 11 nm (2σ) using the Sparrow criterion (Figure S4, Supporting Information).^[^ ^23^ ^]^ With this spatial resolution, two emitters (circled in Figure 1c) separated by 20.8 nm are clearly resolved, as demonstrated in the intensity profile (Figure 1i) taken along two white arrows in Figure 1c. This nanoscale CL imaging of individual quantum emitters with sub‐nm localization precision enables advanced structural characterization. Statistical analysis of another 12 blue emitters shows an average resolution of 13.5 nm (Figure S5, Supporting Information).

### 2.3 Correlative CL Microscopy and Structural Characterization of the Blue Emitter

**Figure 2:**
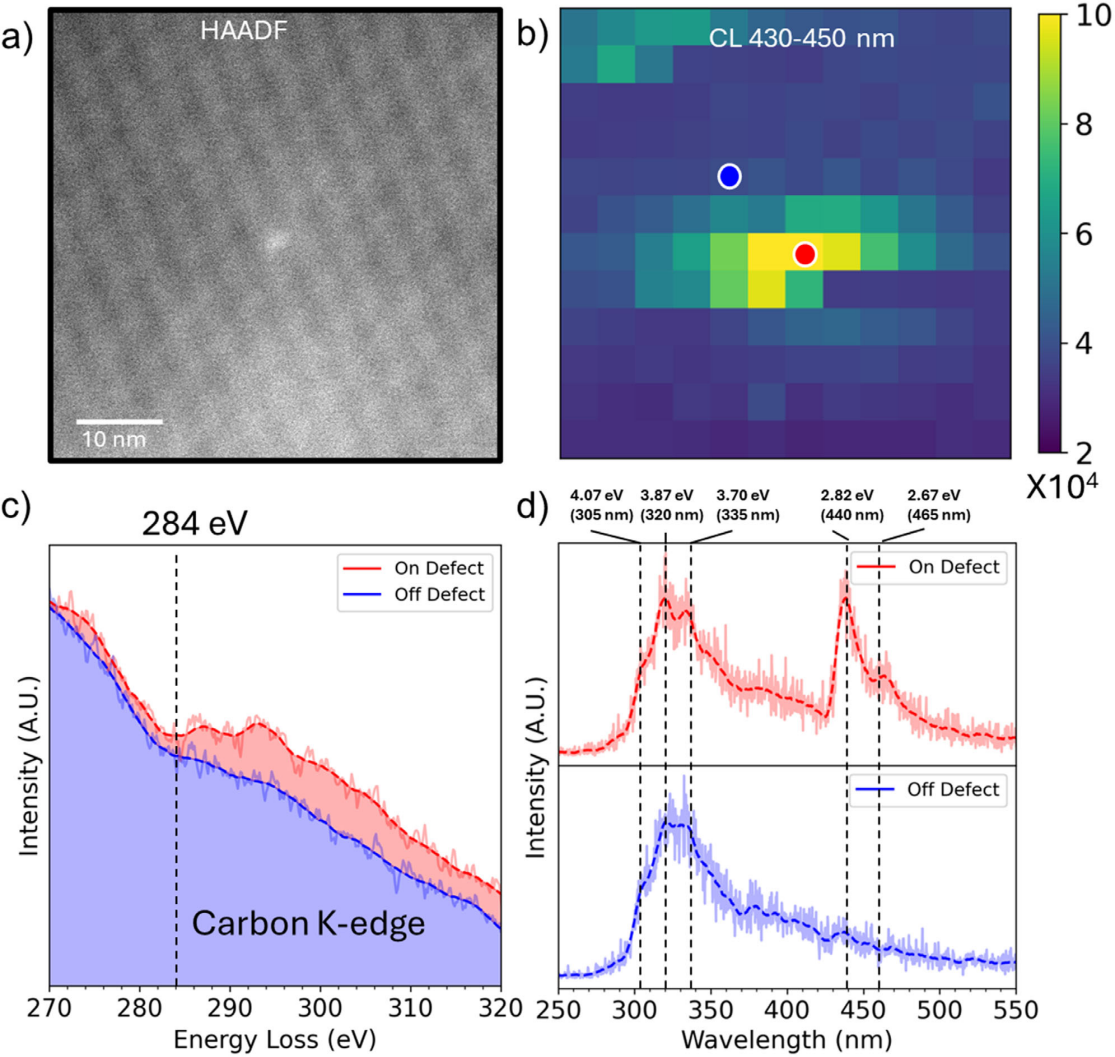
Correlative nanoscale CL microscopy and corresponding STEM‐EELS of the blue emitter. a) HAADF image of a folded area in thin hBN with multiple twist interfaces. The bright contrast in the HAADF image indicates the larger thickness‐mass contrast at the emitter. b) corresponding CL mapping of the 440 nm blue emitter. c) EELS spectra taken on and off the emitter reveal trace amounts of carbon at the same emitter. d) CL Spectra acquired on and off the emitter, showing the 440 nm emission. The 465 nm peak is the PSB of the 440 nm emitter.

Another advantage of the CL enhancement is its ability to maintain a minimum sufficient CL signal for mapping individual emitters, while keeping the sample thin enough for STEM‐based structural and chemical characterizations. **Figure** **2**a shows a HAADF image of a multiple‐fold‐layer hBN with a bright spot in the middle. The unfolded area has a thickness of 10 nm, while the folded area has a thickness of 30 nm. Owing to the CL enhancement provided by the twisted interfaces, STEM‐CL mapping across this area facilitates the collection of sufficient signals. In Figure 2b, the STEM‐CL mapping, reconstructed from signal integration between 430 and 450 nm, reveals an isolated blue emitter corresponding to the bright spot in the HAADF image. As shown in Figure 2d, both CL spectra from on‐ and off‐emitter locations exhibit the UV emission signature, but the marked defective region prominently displays a 440 nm ZPL peak and a 465 nm phonon side band (PSB) peak (also supported in the PL spectrum in Figure S4e, Supporting Information).^[^ ^12^ ^]^ Therefore, the emission center is located at the bright spot in the HAADF image, indicating the blue emitter results from interstitial defects and/or the presence of heavier elements.^[^ ^24^, ^25^ ^]^

To investigate the authentic nature of the defect structure, STEM‐electron energy loss spectroscopy (STEM‐EELS) is performed to obtain chemical information from the same emitter shown in Figure 2a,b. In the EELS spectra on‐ and off‐emitter sites (Figure 2c), the emitter site shows additional carbon signals compared to the off‐emitter site. The double‐peak feature starting from 284 eV indicates the carbon K‐edge and its hybridized bonding.^[^ ^26^, ^27^ ^]^ Elements Si and Ca are also examined, but no signals were observed in EELS spectra as shown in Figure S6 (Supporting Information). Therefore, it can be concluded that the defect with larger thickness‐Z in the HAADF image is carbon‐related. Furthermore, carbon has an atomic number Z of 6, which lies between the atomic numbers of its neighboring elements, boron and nitrogen. Since substitutional carbon defects do not show significant contrast in HAADF images, the blue emitter is likely induced by interstitial carbon‐related defects embedded in hBN.

### 2.4 Atomic Structure Determination of the Blue Emitter

**Figure 3:**
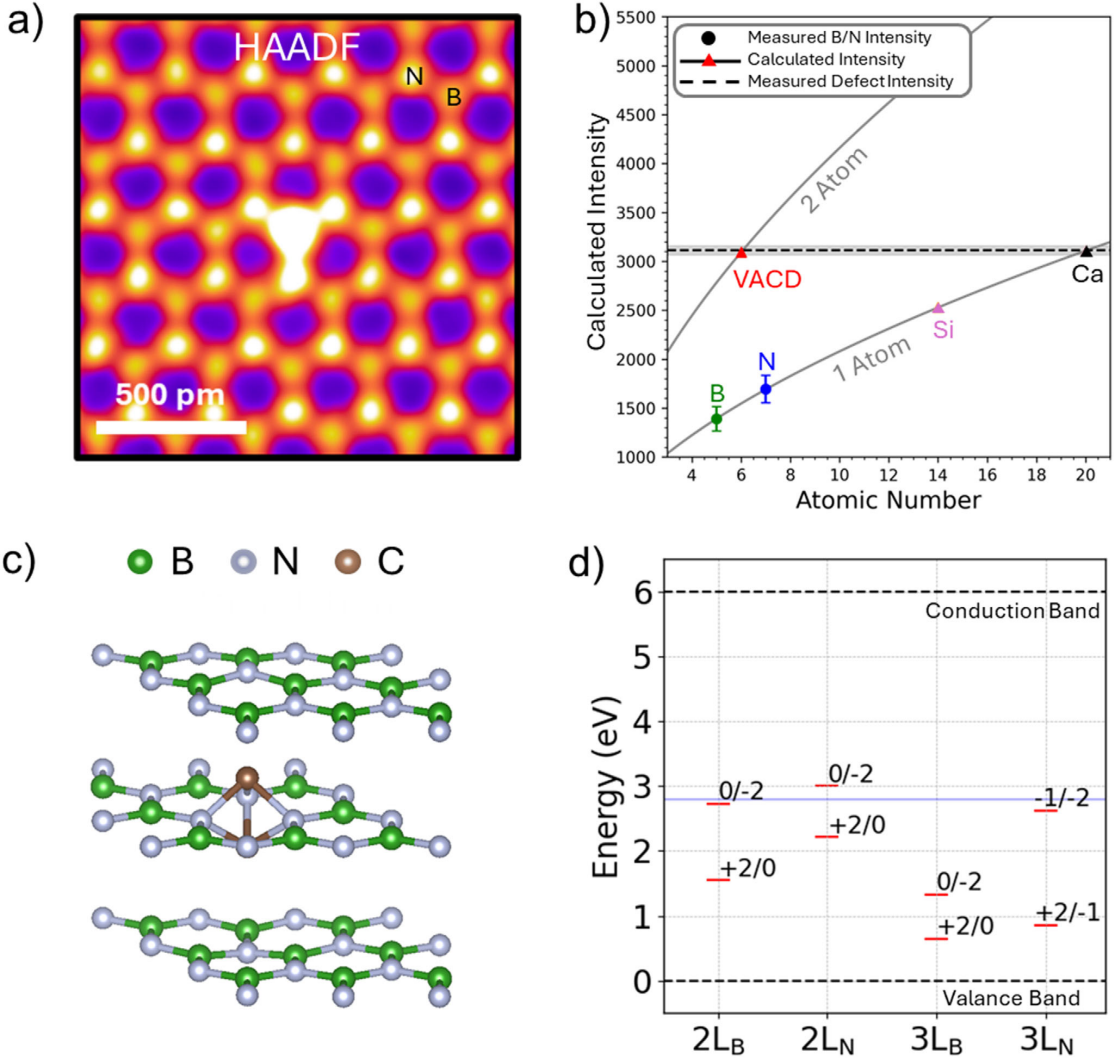
Atomic structure determination of the 440 nm blue emitter. a) Atomic resolution STEM HAADF image of a defect with a bright contrast. b) Quantitative HAADF intensity analysis for various elements and VACD in 3‐monolayer hBN. Only the calculated intensity of VACD matches the experimental intensity indicated by the horizontal dash line. c) Relaxed atomic structure of VACD which is located at the middle layer Boron site. d) Calculated defect levels of VACD at 2‐layer and 3‐layer Boron and Nitrogen sites.

As demonstrated by Zhigulin et al.,^[^ ^22^ ^]^ the quadratic stark effect found under an external electric field parallel to the hBN flake indicates a *D*_3*h*_ point‐group symmetry of the defect due to the absence of a permanent dipole. A VACD structure (split interstitial) is proposed based on the *D*_3*h*_ point‐group symmetry. To verify the atomic structure of the blue emitter, we acquire atomic resolution HAADF images of multiple defects in carbon‐irradiated hBN,^[^ ^28^ ^]^ with an acceleration voltage of 80 kV to avoid knock‐out damage. Several defects including V_B_, V_N_, C_B_C_N_, C_N_C_B_C_N_ are observed as shown in Figure S7 (Supporting Information). None of the observed defects contributes additional bright intensity to the HAADF image as shown in Figure 2a, allowing them to be excluded as potential candidates. **Figure** **3**a presents an atomic‐resolution HAADF image of 3‐monolayer hBN, where the bright and dimmer dots within the honeycomb unit cell correspond to nitrogen and boron atoms, respectively, in the third monolayer. The brightest point defect is located at a dimmer column (boron site in the third monolayer). However, as Figure 3a is a projection of all three monolayers, the point defect will align with a nitrogen site if it resides in the middle layer.

Since the contrast in this HAADF image is Z‐contrast image, the intensity in each atomic column is proportional to Z^x^, where x is between 0 and 2 (Figure S8, Supporting Information).^[^ ^24^, ^25^ ^]^ To quantify the HAADF intensity as evidence of the chemical composition, Figure 3b shows the measured defect intensity and the calculated intensity following the equation in Figure S8 (Supporting Information), with the calculated intensity based on the differentiation between the intensities of boron and nitrogen in a 3‐monolayer hBN. Because this area is a 3‐monolayer with conventional AA’ stacking, the contrast is from the third layer, while underlying boron‐nitrogen pairs contribute indistinguishable signals at each site. The annotations “one atom” and “two atoms” represent defect structures involving either a single atom or a vertically aligned dimer. With boron and nitrogen's intensities confirmed, measured defect site intensity (dash line) intercepts with the “two atoms” at the carbon line, indicating a VACD structure. Other models, such as a single silicon atom, are far away from the measured defect intensity. Thus, the VACD structure is the observed defect in Figure 3a, corresponding to the brighter intensity in Figure 2a.

### 2.5 DFT Simulations of the Blue Emitter

DFT calculations are performed on the vertical dimer defect structure in two‐ and three‐layer hBN structures. We find that the relaxed configuration of the VACD defect is not symmetrically positioned along the hBN layer but slightly protrudes away from the BN surface when the defect is on the surface. However, if the VACD defect is placed in the middle layer of the three‐layer hBN, the defect structure is symmetrically positioned within the hBN layer. Figure 3c shows the relaxed atomic structure of VACD substituting boron in the middle of a 3‐layer hBN. The simulated relaxed structures of different defect configurations and their DOS are shown in Figure S9 (Supporting Information). The observed relaxed VACD structure (Figure S9a, Supporting Information) shows a strong surface effect with a breaking of symmetry when the defect locates at the surface layer. The two carbon atoms in the VACD protrude slightly above the hBN plane after structure relaxation. The topmost carbon atom in B‐site VACD moves toward one of the adjacent nitrogen atoms forming a nitrogen‐carbon bond whereas in N‐site VACD, the carbon atom only leans slightly suggesting a weak interaction with adjacent boron atoms. Consequently, the bright spot in the simulated STEM images for nitrogen site defects as shown in Figure S9 (Supporting Information) is more localized at the N‐site whereas the boron site defect is distributed between the two sites. Meanwhile, the mass center of the atom dumbbell will shift out of the plane due to the presence of an unsymmetrical potential. Therefore, the experimental observations that the VACD preserves point group symmetry suggest that the VACD is embedded within the bulk rather than at the surface. This also explains the long emission stability observed for the buried carbon‐associated emitters in hBN produced by carbon ion irradiation.^[^ ^21^ ^]^

The DOS (Figure S9, Supporting Information) is shifted along the energy axis to align the Valence Band Maximum (VBM) of the defect‐free trilayer hBN at zero, and the vertical dashed line represents the calculated Fermi level (the maximum filled energy level) of the respective defect structure. For hBN, the number of layers has a minor impact on the direct bandgap.^[^ ^29^ ^]^ The calculated Perdew–Burke–Ernzerhof (PBE) bandgap of trilayer hBN is ≈4.3 eV which is less than the experimental bandgap of ≈6 eV. The densities within the bandgap appear due to the introduction of the defect. Although the predicted bandgap is inaccurate, the defect level predictions calculated using the energy differences are accurate^[^ ^30^ ^]^ and comparable to experiments.^[^ ^31^ ^]^ Figure 3d shows the calculated defect transition level energies for the VACD substituting in boron and nitrogen sites in two‐ and three‐layer hBN. Among the different locations of the VACD defect in two‐ and three‐layer hBN, we find defect levels at 2.7, 3.0, and 2.6 eV which are within the range of 2.8 eV within ±0.2 eV which is the typical error between DFT and experiments.^[^ ^30^ ^]^ Further, the defect formation energies for VACD in boron and nitrogen site, same in both bilayer and trilayer hBN, is 157 and 98 meV atom^−1^, respectively, suggesting moderate stability of these defects and supporting the observed long duration of photon emission.

### 2.6 Nanoscale Spatially Controlled Creation of Blue Emitters

**Figure 4:**
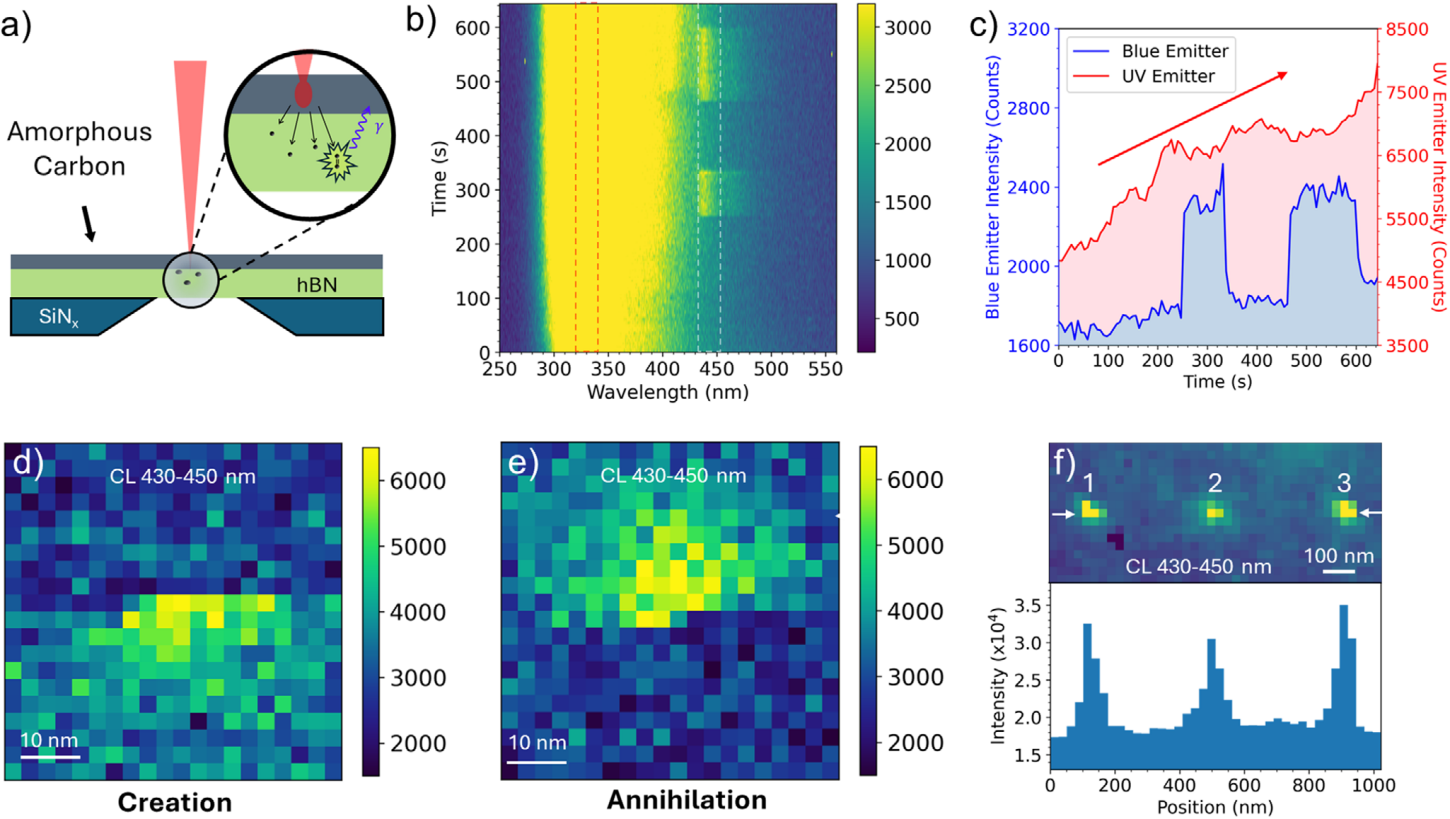
Nanoscale spatially controlled creation of blue emitters on carbon‐coated hBN. a) Schematic diagram of a 5‐nm amorphous carbon layer coated on hBN. b) Time‐dependent CL emission under electron beam irradiation. c) Time‐dependent CL intensity for UV emitters and blue emitters. A continuous increase of intensity is observed for the UV emitter, while an intermittent emission is observed for blue emitters. CL mapping images to show d) creation and e) annihilation of a single blue emitter during mapping. f) (upper) CL mapping of 3 individual blue emitters created at each location by parking e‐beam (≈1 nm diameter, ≈0.2nA) while continuously monitoring the CL spectrum. Once a 440 nm emission appeared, the electron beam was immediately blanked to prevent the formation of additional blue emitters. (lower) intensity profile taken along the two arrows in the upper image.

With the atomic structure of the 440 nm blue emitters understood, we further explore their deterministic creation by irradiating hBN flakes with an electron beam. Although blue emitters can be produced by electron beam irradiation on hBN, as reported previously,^[^ ^12^, ^13^, ^21^, ^22^ ^]^ we find that creating blue emitters solely through electron beam irradiation on pristine hBN is extremely challenging. Recognizing carbon as a key dopant for the VACD blue emitter, we coat hBN with a thin layer of amorphous carbon (≈5 nm) and irradiate it with a high‐current electron beam of ≈0.4 nA as schematically shown in **Figure** **4**a.

Figure 4b shows the time‐dependent CL emission from a twisted sample with the beam (≈1 nm probe size) parked on a stationary spot. While the UV emission at 320 nm remains consistently bright and broad, the blue emitter shows intermittent emission, appearing twice with a specific annihilation duration. Figure 4c shows the line profiles obtained by integrating the signals from both emitters marked in Figure 4b. In comparison, the UV emission is initially present and progressively intensifies during electron beam irradiation, indicating a continuous increase in UV emitter defects caused by the electron beam. The blue emission emerges at 250 s, disappears at 350 s, and reappears at 465 s. Throughout the periods when the emitters are active, the emission intensity remains constant. There is a 100‐s gap between the two emission periods, attributed to the destruction of the emitter by the electron beam rather than photoblinking, as reported by Fournier et al.^[^ ^21^ ^]^ The abrupt formation and annihilation of the blue emitter during beam irradiation suggest that blue emitters are created singularly, in contrast to the large number of UV emitters. The observed blue emitter blinking and UV emission intensity changes might be explained by the beam‐induced transition between the VACD and an in‐plane carbon dimer, which changes the defect electronic level.

To better understand the mechanism behind deterministic creation, we further studied the creation and annihilation processes of the blue emitters. Figure 4d,e show CL mapping of an individual 440 nm emitter which is created and annihilated during mapping. These observations suggest that the creation of individual blue emitters by the electron beam can be monitored through CL and stopped once the emitter is formed. Such observations indicate that the intentional deterministic creation and manipulation of QEs with an electron beam is possible with real‐time feedback. A key challenge in this field is the ability to generate isolated single defects at well‐defined locations. By scanning a confined region while monitoring the CL spectrum, it becomes feasible to create isolated single emitters at desired sites, providing a promising strategy for deterministic emitter placement.

Figure 4f presents STEM‐CL mapping (430–450 nm) of a 1 × 3 array of blue emitter sites created by focused electron beam irradiation on carbon‐coated hBN (see Experimental Section for details). To improve spatial precision and favor the formation of isolated single defects, we used a focused electron beam (≈1 nm probe size) in a parked‐beam mode on carbon‐coated hBN, while monitoring CL emissions in real time. The electron beam was immediately turned off once a 440 nm emission peak appeared in the CL spectrum at each targeted site. However, when we attempted to scan the resulting emitter array with high‐resolution pixel sizes (2–3 nm), we often observed unintended effects: additional emitters were unintentionally generated like the creation in Figure 4d, or existing emitters were erased like the annihilation in Figure 4e, making it challenging to preserve and visualize the intended pattern. To avoid these issues, we used a coarser pixel size of 25 nm for post‐creation CL mapping. This approach minimized scan‐induced modification during imaging and successfully preserved the emitter array, as shown in Figure 4f.

Although the emitters appear as ≈50 nm spots due to the coarse mapping resolution, each spot likely corresponds to a single emitter. The intensity profile of the three emitters (lower panel, Figure 4f) shows comparable emission strengths, supporting the interpretation that they are single defects rather than coincidental aggregates of multiple emitters. Figure S10 (Supporting Information) displays a CL spectral line scan along two arrows in Figure 1f, confirming that all three emitters exhibit similar blue emissions. Since all emitters were created using the CL‐monitored feedback protocol, it is more reasonable to attribute the consistent emission to individual defects with similar characteristics, rather than clusters of identical number of blue emitters. However, confirmation of single‐photon emission requires g^(2)^(t) measurements. These results highlight the value of real‐time CL monitoring for achieving spatially controlled creation of the desired emitter. With continued improvements in CL sensitivity and beam stability, it may become possible to generate even smaller, more spatially defined single‐photon emitters.

## 3 Conclusion

In this work, the direct imaging of individual quantum emitters at nanometer resolution with sub‐nm localization precision is achieved using STEM‐CL in thin twisted hBN. This high spatial resolution CL imaging capability enables us to determine the atomic structure of the 440 nm blue emitter to be vertically aligned carbon dimer, providing the critical atomic insights about the relationship between atomic structure and emission properties. The demonstrated CL imaging capability at nanometer resolution also opens a range of possibilities for the study of electronic structure, quantum coherence, and lifetime of quantum emitters at the nanoscale. Thus, by advancing our understanding of emitter structures using cathodoluminescence mapping, this research paves the way for the precise engineering of quantum emitters, significantly impacting the development of advanced quantum information technologies.

## 4 Experimental Section

### Synthesis of Exfoliated hBN on TEM Grids

Pristine hBN single crystals in Figure 1b and Figure S1 (Supporting Information) were from Japan. Pristine hBN single crystals in all other figures were purchased from HQ graphene (Europe). Exfoliated hBN flakes on SPV‐224PR‐MJ tape were transferred onto the polydimethylsiloxane (PDMS) film. Then, the hBN flakes on the PDMS film were stamped onto SiN_x_ TEM grids with holes.

### Scanning Transmission Electron Microscopy

The high‐resolution characterizations including imaging, EELS are performed on a Thermo Fisher Spectra 300 in aberration‐corrected STEM mode. To avoid knock‐on damage on hBN, STEM/TEM are operated in 80 kV to minimize the effect. The optimized probe size at 80 kV is 0.12 nm, which is smaller than the B‐N dumbbell distance of 0.144 nm in hBN. EELS was acquired using a Gatan Image Filter Quantum ER System with a dual channel. The twisted angle is measured by the selected area diffraction pattern in a TEM mode.

### STEM‐CL and CL Hyperspectral Imaging System

The STEM‐CL was performed on the Quantum Emitter Electron Nanomaterial Microscope (QuEEN‐M), a Thermo Fisher Spectra‐300 probe corrected STEM, at Argonne National Laboratory. The QuEEN‐M has a large pole‐piece gap of 6.3 mm, enabling to host a parabolic mirror of the Attolight M¨onch CL module, which is decoupled from the TEM sample holder. A specially designed Mel‐built cryo CL holder was used for CL acquisition. The microscope was configured with a gun lens size of 1 and a spot size of 3, leading to a typical e‐beam current of 0.1–0.5 nA. An Oxford Newton EMCCD is used to acquire CL spectra. For CL hyper‐mapping, a dwell time ranging from 0.1 to 5 s was used for each pixel.

### Masked‐Carbon‐Ion‐Implantation (MCI)

Carbon deceleration mask preparation The 400 Mesh TEM copper grids with carbon film were used as the substrate to grow the carbon deceleration mask. For specific carbon mask thickness, carbon deposition through chemical vapor deposition were used with a typical thickness variation of ±10 nm. For thick samples, carbon film supported on single holed TEM grids (CFGA1500‐Cu‐ET) purchased from Electron Microscope Sciences were used. The grids have an aperture with a diameter of 1500 µm and the claimed thickness of 25–30 nm (One mask was tested by the EELS measurement in TEM, resulting in a thickness of 26 nm, confirming the claimed value). In a typical MIC treatment, two grids were stacked together to obtain an effective thickness of 50 nm.

The masked‐carbon‐ion‐implantation was done with the ion accelerating source of the Nuclear Science and Engineering division of Argonne National Laboratory. The carbon ion was obtained by ionization of carbon oxide molecules. The ions were separated by the acceleration field, where an acceleration voltage 40 kV was applied. Then the carbon ions were focused and guided on the sample grids by magnetic lenses. During the ion implantation, the carbon mask was placed in front of the sample grid to achieve the desired ion penetration depth in the hBN flake. The typical fluences of the carbon ions applied in the treatment were: 2.5 × 10^14^.

### *g* ^2^ Measurement

The second‐order correlation function 𝑔^2^(t) of the quantum emitters was measured using a custom‐designed upright confocal optical microscope. The main structural components of the microscope were sourced from *Thorlabs* (DIY *Cerna* Components and the cage system). A 100× *Mitutoyo* Plan Apo HR infinity‐corrected objective was employed to both focus the excitation laser (405 nm CW laser light generated from a diode laser and filtered by a 405–10 band‐pass filter before sending into the microscope) onto the sample and collect the resulting photoluminescence (PL) from the quantum emitter. The collected PL was first directed, via a high‐reflectivity mirror mounted on a DC brushless motorized stage, into a Kymera 328i spectrograph equipped with a 500 lines mm^−1^ grating blazed at 500 nm for spectral analysis. Subsequently, the light was coupled into a custom‐built 50:50 multimode fiber beamsplitter (FC/APC connectors) through a reflective fiber coupler coated with UV‐enhanced aluminum, which evenly distributed the photons to two single‐photon avalanche diodes from Micro Photon Devices (MPD). Time‐correlated single‐photon counting was performed using a MultiHarp module from *PicoQuant*, and the temporal photon correlation was analyzed using the QuCoa (*PicoQuant*) software package.

### Nanoscale Spatially Controlled Creation of Blue Emitters

A thin layer of Carbon film (<10 nm) is coated on the surface of hBN by carbon rod coating. The irradiated spot is a 1 × 3 array. The CL displayed is acquired with the electron probe of 0.2 nA and 1s per pixel.

### DFT Calculations

All the density functional theory calculations are performed with the Vienna Ab Initio Simulation Package (VASP) code^[^ ^32^, ^33^, ^34^ ^]^ using the PBE exchange correlation functional^[^ ^35^ ^]^ within the GGA approximation. To account for the van der Waals interactions between the hexagonal BN layers, the nonlocal vdw‐DF functional was used.^[^ ^36^ ^]^ The MaterialsProject^[^^]^ recommended pseudopotentials with projector augmented wave (PAW) formalism are used for B, C, N which include 3, 4, and 5 valence electrons respectively. A cutoff energy of 500 eV and an automatic k‐point mesh with 50 kpoints per inverse angstrom are used for all calculations. Charged defect calculations with −2, −1, 0, +1, and +2 charges were performed to the supercell with correction methodology^[^ ^39^ ^]^ to predict the defect transition levels by adding or removing the number of electrons in the system. The vacuum thickness for neutral and charged calculations is maintained to be at least 20 and 30 Å, respectively, to avoid interactions between periodic images of the defects along the z‐direction. The simulated STEM images are driven by the Multislice algorithm as implemented in the abTEM package.^[^ ^40^, ^41^ ^]^

## Conflict of Interest

The authors declare no conflict of interest.

## Author Contributions

H.H. and M.H. contributed equally to this work. M.H., T.G., B.D., and J.W. conceived the project and designed the experiments. H.H., M.H., P.T., T.G., and J.W. conducted the TEM experiments. M.H. and B.D. conducted optical experiments. M.H. and W.C. performed masked‐carbon‐ion‐implantation. V.K. and M.C. carried out DFT simulations. H.H. and K.Y. coated carbon film and performed deterministic creation. H.H., T.G., J.Z., and J.W. wrote the manuscript. All authors discussed the results and commented on the manuscript.

## Supporting information



Supporting Information

## Data Availability

The data that support the findings of this study are available from the corresponding author upon reasonable request.
