# Supplementary material for: Nanometer Resolution Structure‐Emission Correlation of Individual Quantum Emitters via Enhanced Cathodoluminescence in Twisted Hexagonal Boron Nitride
Source: Adv Mater. 2025 Jul 24;37(41):e01611. doi: 10.1002/adma.202501611 (PMC12531738; doi:10.1002/adma.202501611)
Supplement: Supplementary file 1 — Supporting Information [file ADMA-37-e01611-s001.docx]

**Supplementary Information**

**Nanometer Resolution Structure-Emission Correlation of Individual Quantum Emitters via Enhanced Cathodoluminescence in Twisted Hexagonal Boron Nitride**

Hanyu Hou^1,2†^, Muchuan Hua^1†^, Venkata Surya Chaitanya Kolluru^1^, Wei-Ying Chen^3^, Kaijun Yin^2^, Pinak Tripathi^2^, Maria K.Y. Chan^1^, Benjamin T. Diroll^1*^, Thomas E. Gage^1*^, Jian-Min Zuo^2*^, Jianguo Wen^1*^

**Affiliations:**

^1^Center for Nanoscale Materials, Argonne National Laboratory, 9700 S. Cass Avenue, Lemont, Illinois 60439, the United States.

^2^Department of Materials Science and Engineering, Materials Research Laboratory, University of Illinois Urbana Champaign, 1304 W. Green St. MC 246, Urbana, Illinois 61801, the

United States.

^3^Nuclear Science and Engineering, Argonne National Laboratory, 9700 S. Cass Avenue, Lemont, Illinois 60439, the United States.

^†^These authors contributed equally to this work.

^*^Corresponding authors: Benjamin T. Diroll, (bdiroll@anl.gov), Thomas E. Gage (tgage@anl.gov), Jian-min Zuo (jianzuo@illinois.edu), Jianguo Wen (jwen@anl.gov)

Supplementary Information includes:

SI-Table 1 to SI-Table 2

SI-1 to SI-10

**Supplementary Information SI-Table 1**

Detailed Sample information


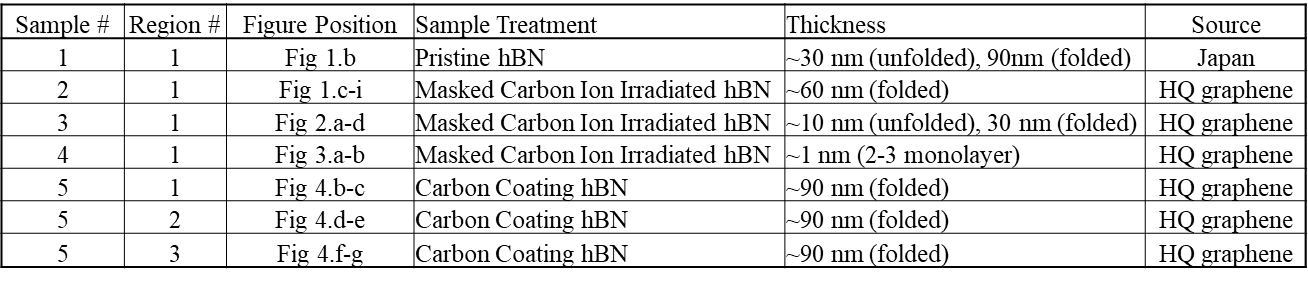


**Supplementary Information SI-Table 2**

Counts and acquisition details

**
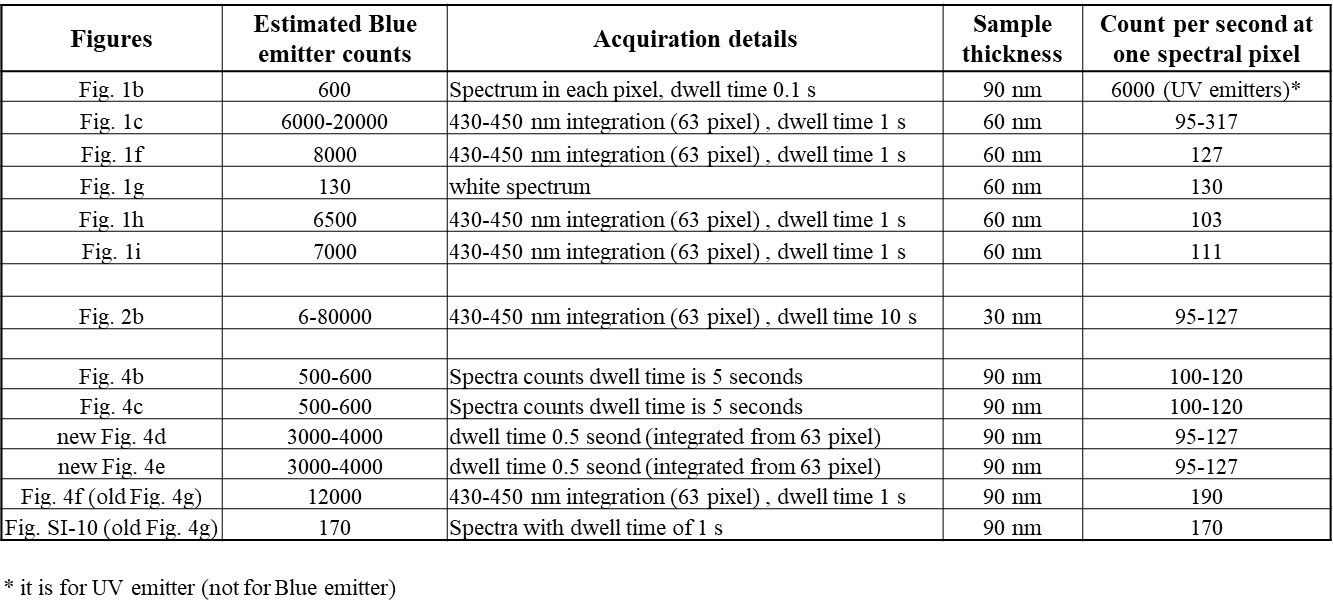
**

After excluding the obvious bright spots in Fig. 1c—which correspond to clusters of blue emitters—the typical emission from isolated single blue emitters is approximately 100–200 counts.

**Supplementary Information SI-1**

Temperature-dependent CL spectra from twisted hBN


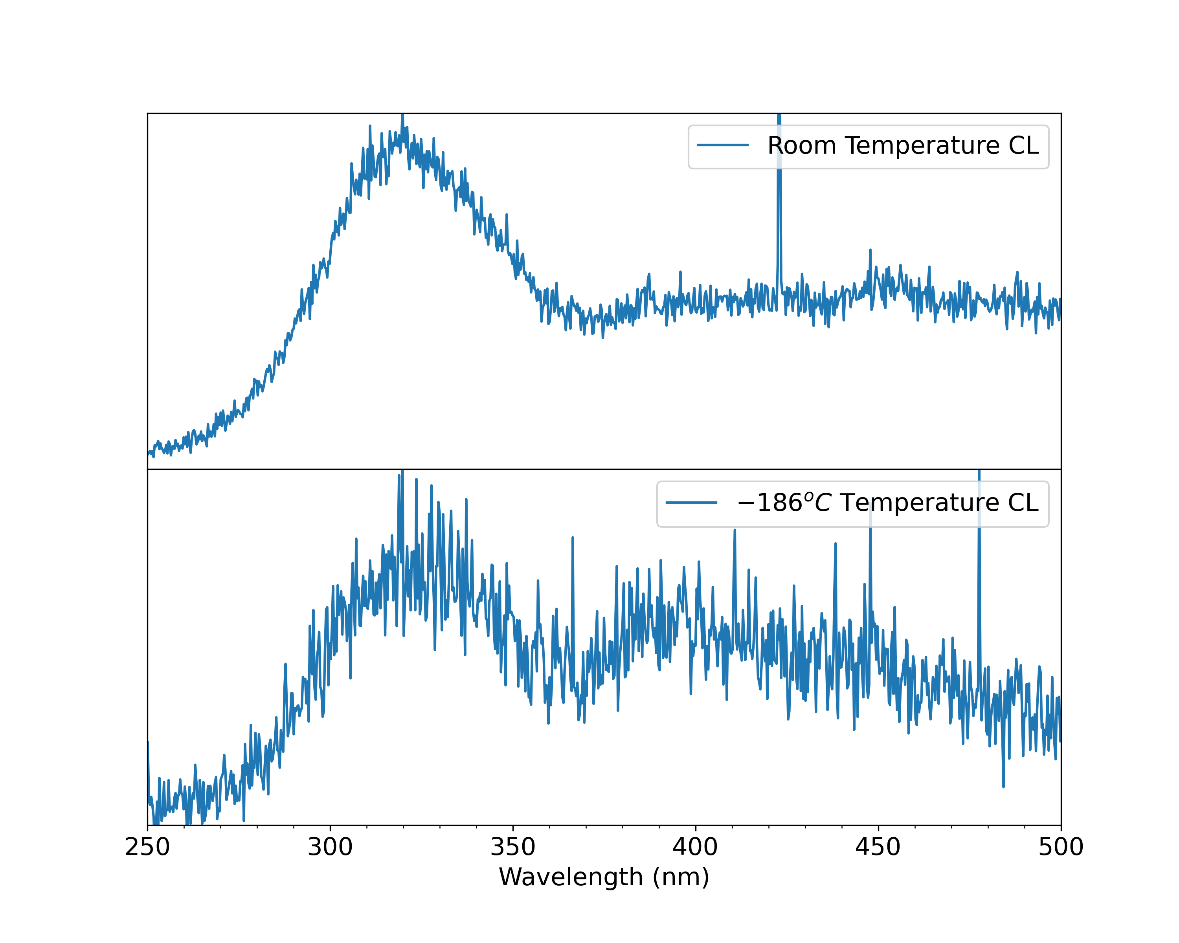


**Fig. SI-1**: CL spectra of hBN (from Japan) taken at room temperature and -186°C. A 300 nm ZPL can be observed at -186°C.

**Supplementary Information SI-2**

Twist angle and thickness in the folded hBN

**
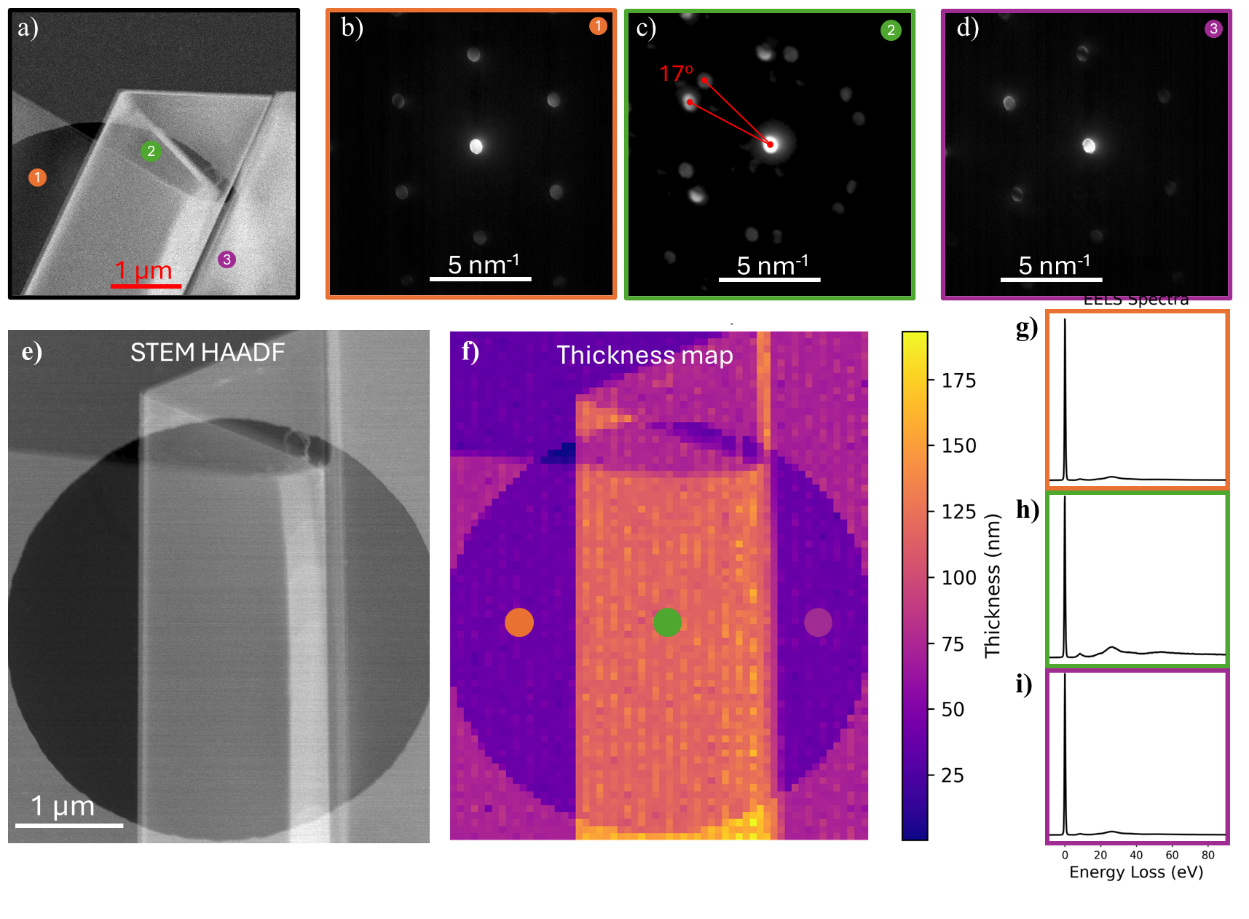
**

**Fig. SI-2** a) HAADF image; b)-d) Nanobeam electron diffraction patterns showing 17° degree twist angle. e) HAADF image and corresponding f) thickness map using STEM-EELS. g)-i) Low-loss EELS spectra from 1L, 3L and 1L showing thicknesses of 30 nm, 90 nm, and 30 nm.

**Supplementary Information SI-3**

Hyperspectral CL mapping of UV and 440 blue emitters in hBN

**
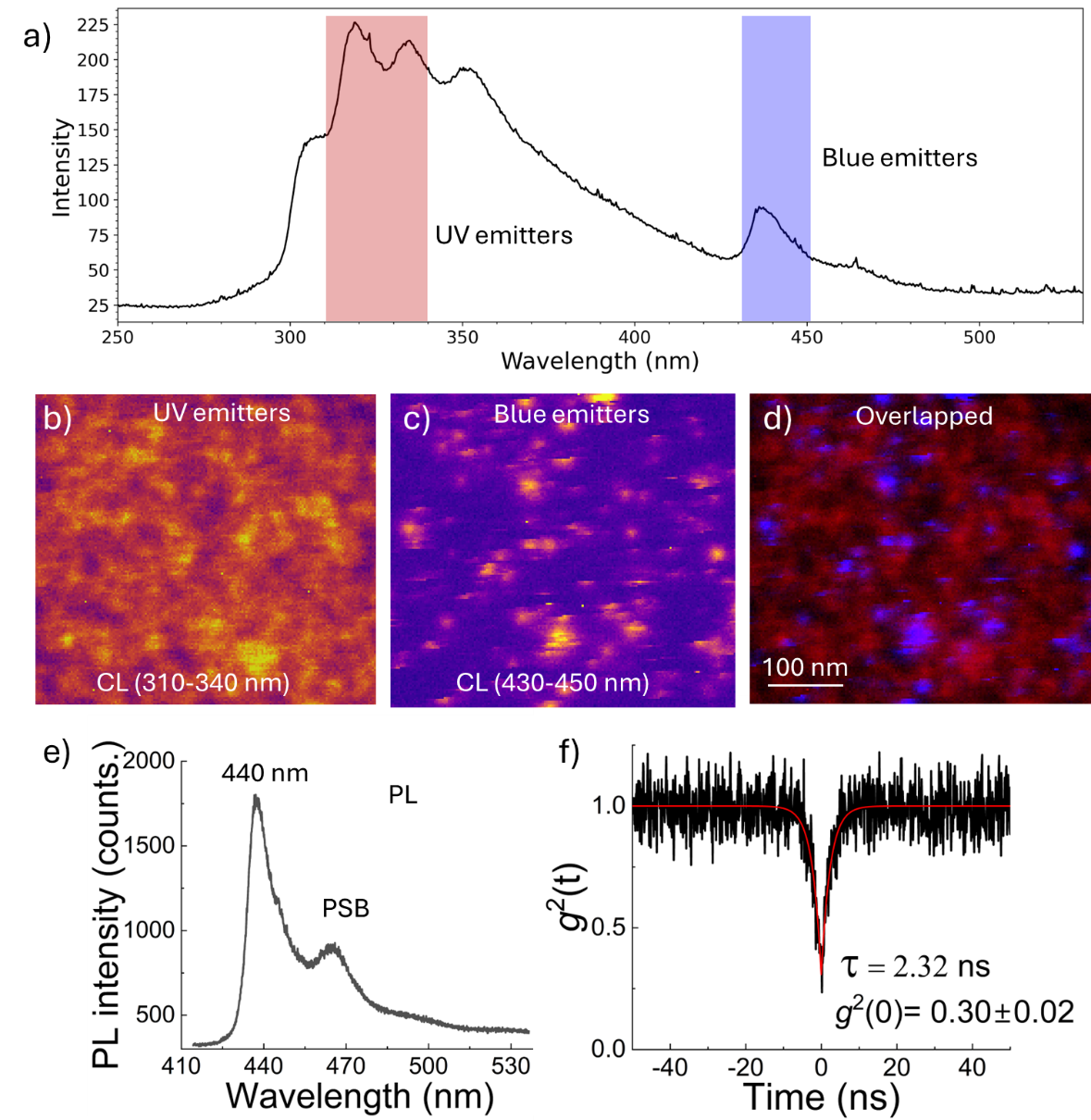
**

**Fig. SI-3** CL mapping of UV and blue emitters in carbon ion irradiated hBN. a) Integrated CL spectrum showing UV and blue emissions; b) CL mapping of UV emitters in hBN. c) CL mapping of blue emitters. d) Overlapped map showing the distribution of UV and blue emitters.

**Supplementary Information SI-4**

Super Resolution Microscopy Analysis

Since the emitters in hBN are single-photon sources, their emission can be represented by a point spread function. This enables the use of super-resolution microscopy to analyze these quantum emitters through Gaussian fitting. Here, we applied this technique to one of the high-quality CL mappings on the creation of the emitter. We applied mirror symmetry on the semicircle of the CL emitter as shown below:


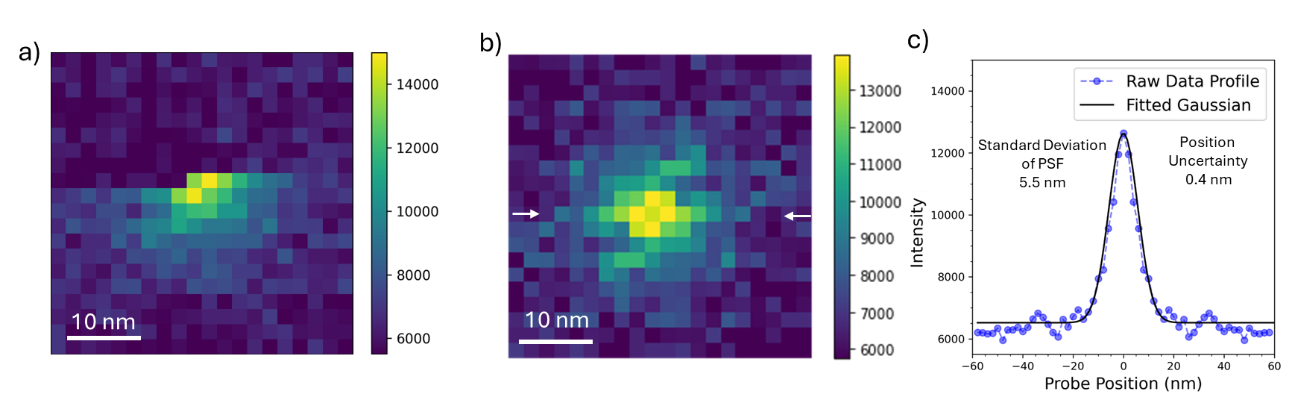


This operation better help to finish the gaussian fitting without changing sigma along x direction, and while we assume the feature is isotropic, σ_x_ and σ_y_ should be very similar.

We then apply gaussian point spread function (PSF) fitting to the intensity map of the emission. The fitted σ_x_ is 5.75 nm and σ_y_ is 5.30 nm. These standard deviations of the PSF are primarily limited by the charge carrier diffusion length in this extreme case, because the aberration-corrected electron beam with a probe size of 0.2 nm under 80kV is much smaller than the scanning scale (2 nm per pixel).

To define the precision of the emitter’s position in super resolution microscopy, the equation below describes the uncertainty^1^:

$$\sigma=\sqrt{\frac{s^{2}+a^{2}/12}{N}+\frac{8\pi s^{4}}{a^{2}}\frac{b^{2}}{N^{2}}}$$

Here, s is the standard deviation of the PSF; N is the total number of photon counts; a is the pixel size and b is the noise in the background. To convert measure CL intensity to photon counts, we thereby using the conventional equation as shown below to calculate:

$$N=\frac{I}{G\cdot QE}$$

N is the photon of collected, I is the intensity of collected, G and QE are the gain and quantum efficiency of the detector. Because we used an electron-multiplying charge coupled device (EMCCD), with the selected electron multiplier gain of 200 (0-255), the measured electron amplification is near 600 under exponential approximation according to the Andor Newton EMCCD manual. There is another 4 times gain from the detector. These induced 2400 times signal amplification in total. The quantum efficiency according to the manual near 440 nm is 70%. Therefore, we could measure the photon counts of 188. After plugging back into the equation, the calculated uncertainty of emitter position is 0.42 nm. Considering the reasonable amplification of EMCCD is ~100 times, the measured photon counts is 1128 and the uncertainty of emitter position is 0.21nm. Therefore, the position uncertainty is below one nanometer.

**Supplementary Information SI-5**

**Statistical analysis of resolution and localization precision for blue emitters**


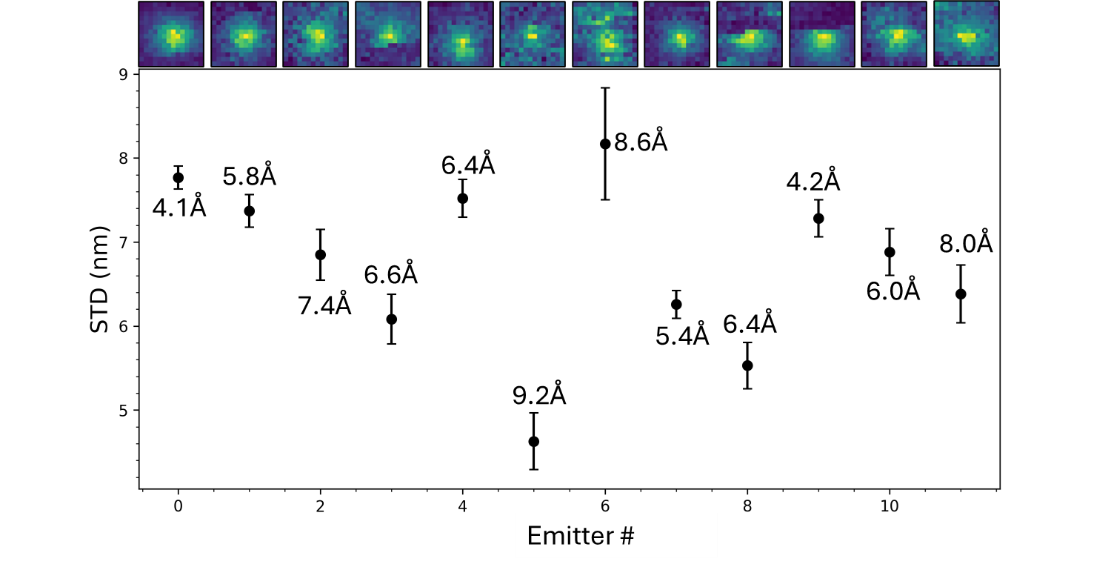


**Fig. SI-5**: Statistical analysis of resolution and localization precision for 12 other blue emitters. Note: resolution = 2*STD. The number in the figure represents the localization precision. The error bar represents the fitting error for point spread function STD. The average STD for 12 emitters is 6.76 nm.

**Supplementary Information SI-6**

EELS information for Si and Ca

**
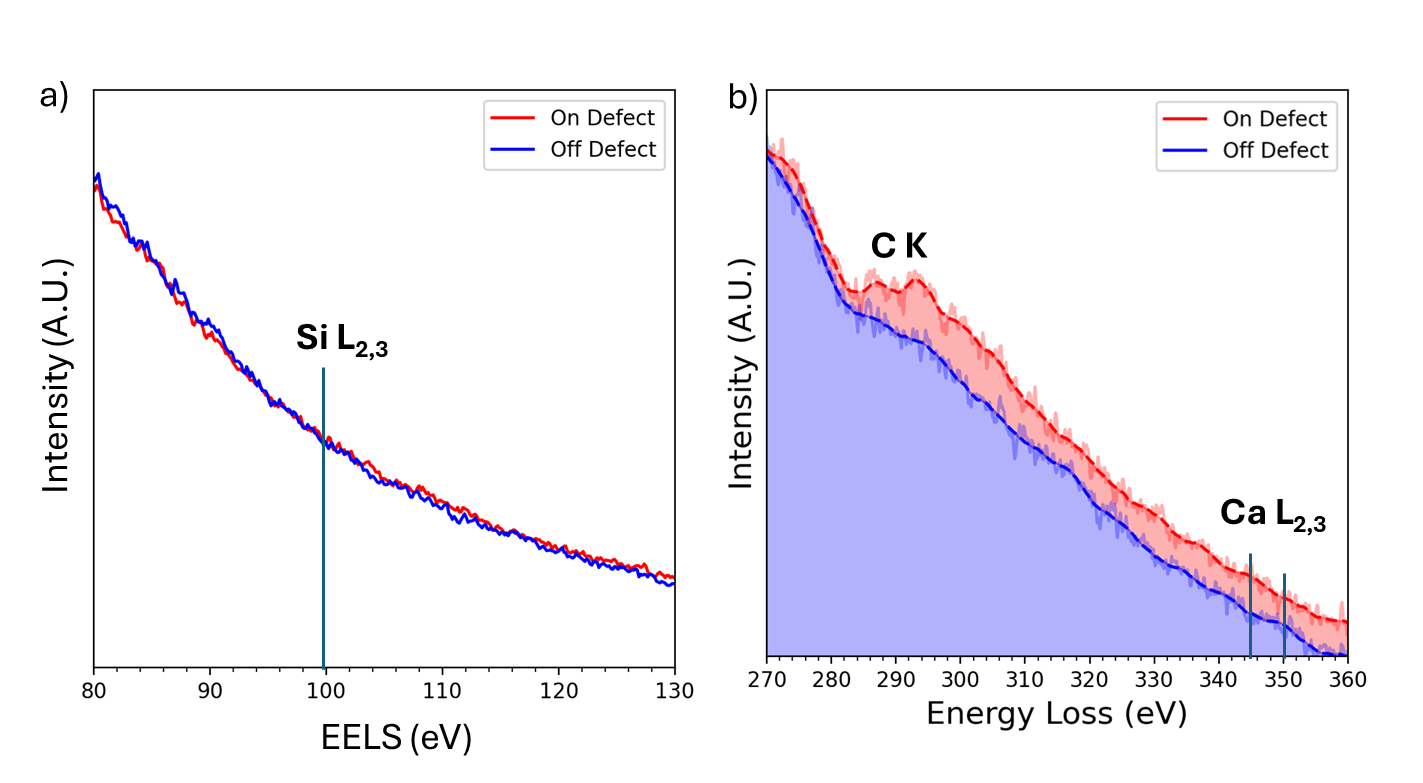
**

**Fig. SI-6** EELS spectra from on- and off-emitter showing no extra Si and Ca at the emitter.

**Supplementary Information SI-7**

Atomic resolution HAADF images of several defects

in carbon-ion irradiated hBN

**
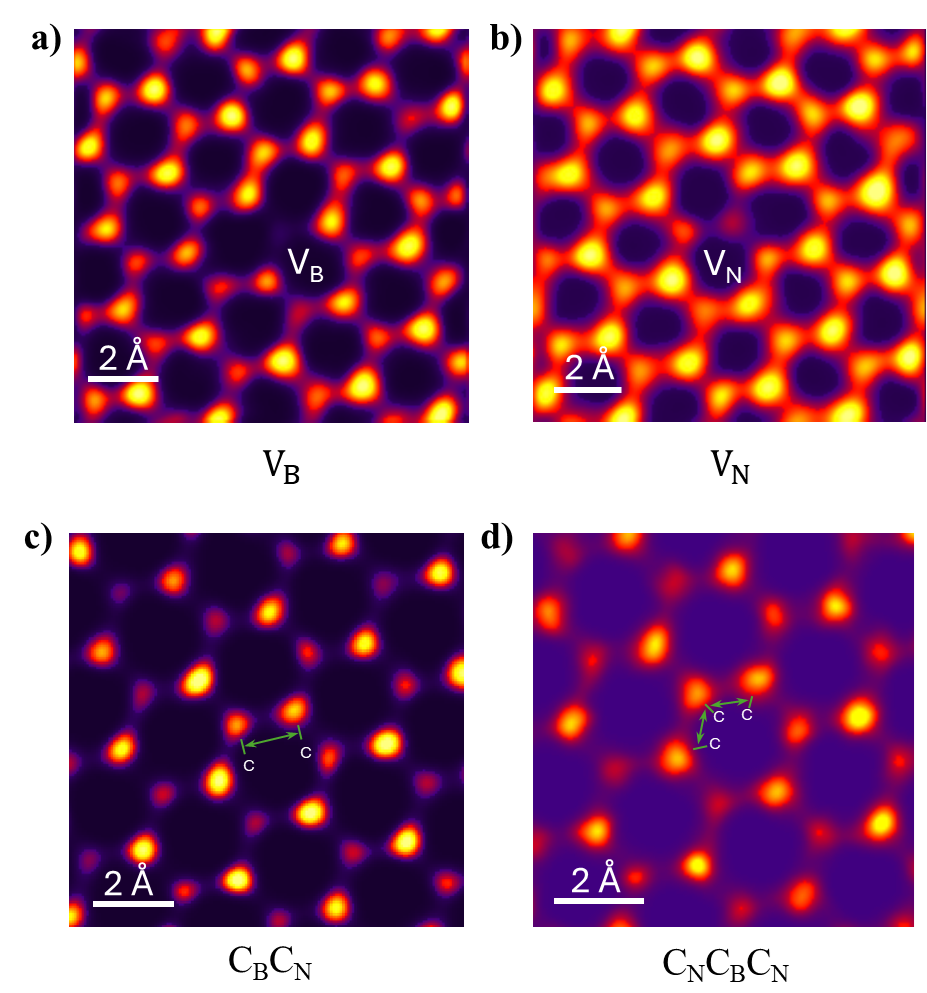
**

**Fig. SI-7** HAADF images showing several typical defects in carbon-ion irradiated hBN.

**Supplementary Information SI-8**

Formula for quantitative HAADF intensity

$$I=\sum_{i} \frac{\alpha}{\lambda_{i}}t_{i}{Z_{i}}^{x}+I_{0}$$

The intensity *I* accounts for contributions from atoms in a column, using a thin thickness approach, and includes the dark counts as *I*_0_. In this equation, α is the adjusted proportionality constant that encapsulates experimental conditions such as incident beam current, detector gain, and other instrumental factors. This value remains constant under the same imaging conditions. Each atom in the column contributes to the total STEM intensity based on its atomic number *Z*_i_, the thickness *t*_i_ (here is the number of monolayers), and the electron mean free path λ_i_. The atomic number *Z*_i_ is raised to the power of *x*, an exponent ranging from 0 to 2, determined by the detector collection angle. This exponential term highlights the Z-dependent STEM imaging.^2,3^

**
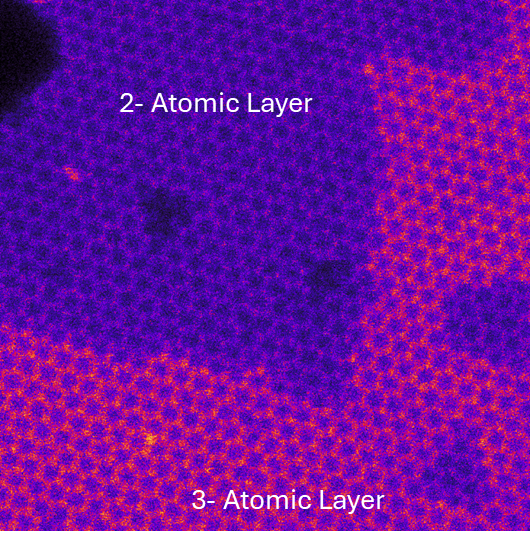
**For instance, *I*_0_​ is measured from the vacuum region (located in the upper left corner). Individual atomic columns are initially identified using a blob detection algorithm. To ensure accuracy, the intensity for each column in 2-monolayer or 3-monolayer regions is averaged over multiple atomic columns of the same type. In the 2-monolayer region, 2 adjacent atomic columns show the same intensity due to the AA’ stacking in hBN. The intensities of boron and nitrogen in the third monolayer can be determined by subtracting the intensity of the 2-monolayer region from that of the 3-monolayer region.

**Fig. SI-8** Original HAADF Image (without any image process) showing 3-monolayer, 2-monolayer, and vacuum. Boron and nitrogen intensities are calibrated using the internal calibration from these three areas.

**Supplementary Information SI-9**

**
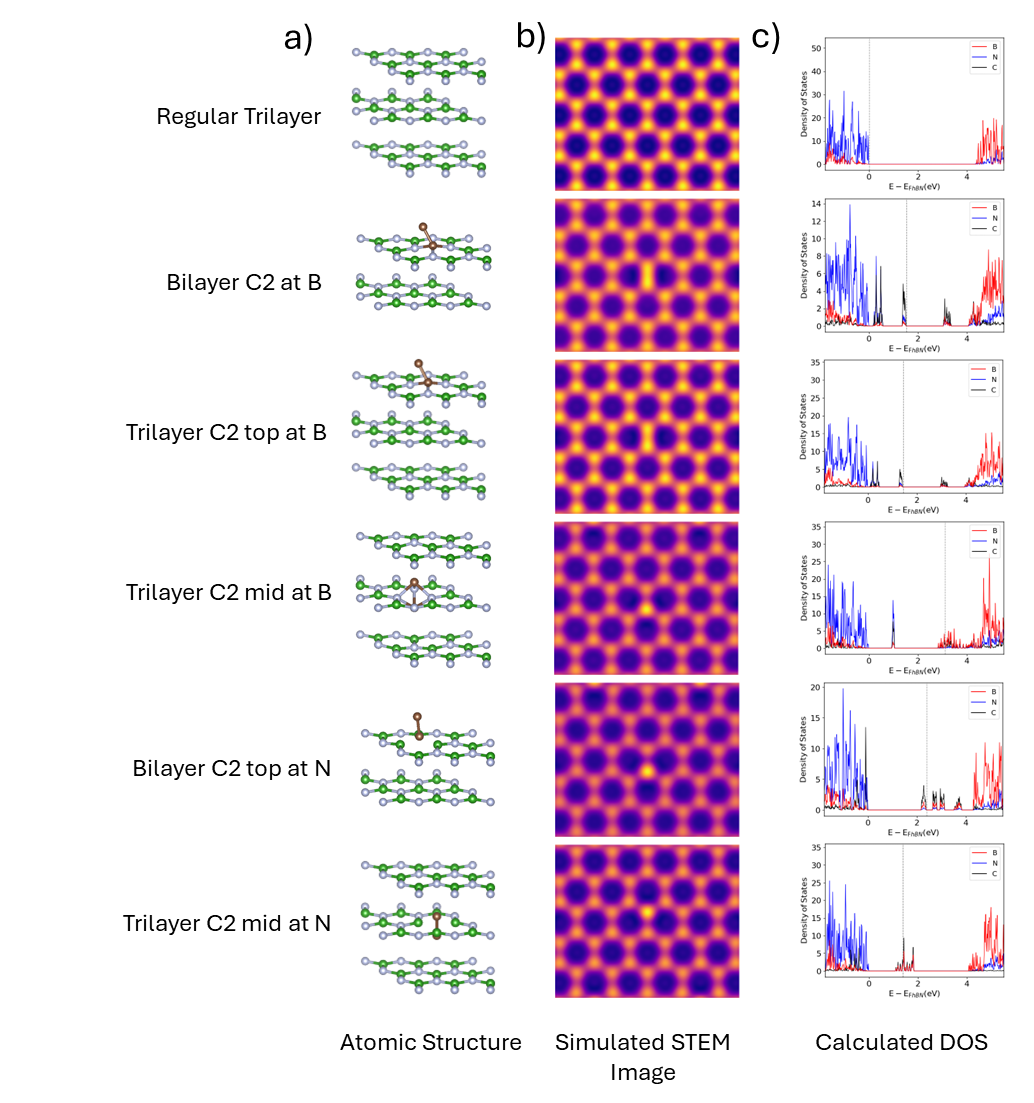
**

**Fig. SI-9** Relaxed atomic configurations from DFT calculations for various VACD structural models, simulated STEM images, and calculated density of states (DOS). The vertical dashed line separates the filled and unfilled states.

**Supplementary Information SI-10**


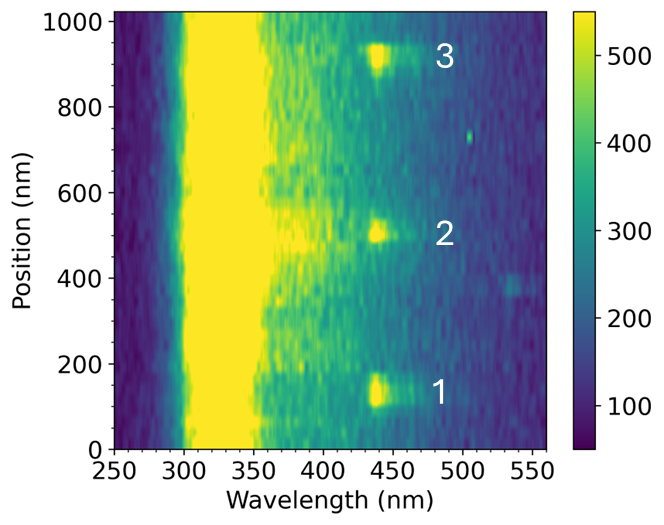


**Fig. SI-10** CL spectral line scan along two arrows in Fig. 4f, showing three blue emitters with similar emission features.

**References:**

1. Pertsinidis, A., Zhang, Y. & Chu, S. Subnanometre single-molecule localization, registration and distance measurements. *Nature* **466**, 647–651 (2010).

2. Zuo, J. M. & Spence, J. C. H. *Advanced Transmission Electron Microscopy*. (Springer, New York, NY, 2017). doi:10.1007/978-1-4939-6607-3.

3. Williams, D. B. & Carter, C. B. *Transmission Electron Microscopy*. (Springer US, Boston, MA, 2009). doi:10.1007/978-0-387-76501-3.
